# Supplementary material for: Suitability of resampled multispectral datasets for mapping flowering plants in the Kenyan savannah
Source: PLoS One. 2020 Sep 22;15(9):e0232313. doi: 10.1371/journal.pone.0232313 (PMC7508412; doi:10.1371/journal.pone.0232313)
Supplement: S1 Table — (PDF) [file pone.0232313.s012.pdf]

Table S1: Photos of the representative plants in the various flowering functional groups used for generating the flower maps in the Mwingi study site. These photos were taken by the lead author during field surveys curried out in January 2014 and February 2013.

Functional group	Species	Photo
**White flowers**1	*Terminalia brownee*	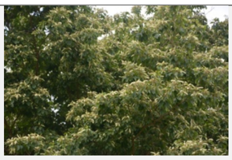
**White flowers**2	*Acacia tortilis*	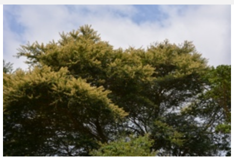
**Yellow flowers**	*Acacia nilotica*	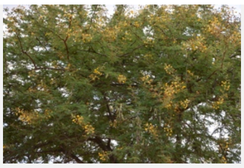
**Crops**1	*Zae mays*	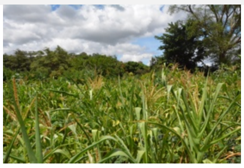
**Crops**2	Millet	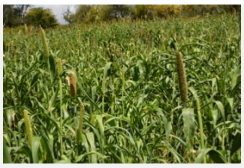
**Flowering fobs**	*Ipomea vatke*	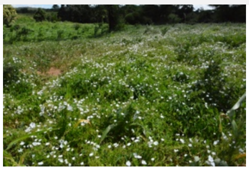
**Senesced trees**	Senesced trees	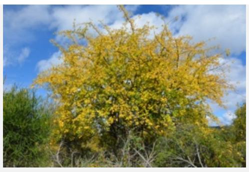

* crops1 and crops2 both belong to the functional group *'crops'* while white flowers1 and white flowers2 both belong to the functional group *'white flowers'*.
